# Supplementary material for: Senolytic Combination Treatment Is More Potent Than Single Drugs in Reducing Inflammatory and Senescence Burden in Cells from Painful Degenerating IVDs
Source: Biomolecules. 2023 Aug 16;13(8):1257. doi: 10.3390/biom13081257 (PMC10452201; doi:10.3390/biom13081257)
Supplement: Supplementary file 1 [file biomolecules-13-01257-s001.zip › Supplementary Tables.pdf]

Table 1 - Characteristics of the Painful Degenerate Donors Utilized in the Study (-). (ICC): Immunocytochemistry of monolayer culture including p16^{INK4a}, Ki-67 and Caspase-3. Immunofluorescence of pellet culture for p16^{INK4a}, Ki-67 and Caspase-3. (RT-qPCR): Real-time Quantitative Polymerase Chain Reaction, (ELISA): Enzyme-linked immunosorbent assays, (DMMB): Dimethyl methylene blue (DMMB) assays.

Donor	Age	Sex	ICC	IHC	RT-qPCR	ELISA	DMMB
1	32	M	-		-	-	
2	65	F	-		-	-	
3	66	M	-		-	-	
4	47	F	-		-	-	
5	61	F	-	-	-	-	-
6	55	F	-	-	-	-	-
7	45	M	-	-	-	-	-
8	35	F		-	-	-	-
9	67	M		-	-	-	-
10	43	M		-	-	-	-
11	56	M		-	-	-	-

Table 2 - qRT-PCR Primer Sequences

Target	Forward Primer Sequence	Reverse Primer Sequence	Reference
BDNF	5'-TAACGGCGGCAGACAAAAAGA-3'	5'-GAAGTATTGCTTCAGTTGGCCT-3'	Krock et al., 2017
CCL2	5'-GCATGAAAGTCTCTGCCG-3'	5'-GAGTGTTCAAGTCTTCGGA-3'	Sandell et al. 2008
CCL5	5'-GAAGGTCTCCGCGGCAGCC-3'	5'-CTGGGCCCTTCAAGGAGCGG-3'	Sandell et al. 2008
CCL7	5'-CACTTCTGTGTCTGCTGCTCAC-3'	5'-GTTTTCTTGTCCAGGTGCTTCATA-3'	Wang et al. 2011
CCL8	5'-GCCTGCTGCTCATGGCAGCC-3'	5'-GCACAGACCTCCTTGCCCCG-3'	Sandell et al. 2008
CXCL10	5'-GTGGCATTCAAGGAGTACCTC-3'	5'-TGATGGCCTTCGATTCTGGATT-3'	Sandell et al. 2008
CXCL8/ IL-8	5'-TCCTGATTTCTGCAGCTCTG-3'	5'-GTCTTTATGCACTGACATCTAAGTTC-3'	Cherif et al. 2019
G-CSF	5'-GAGCAAGTGAGGAAGATCCAG-3'	5'-CAGCTTGTAGGTGGCACACTC-3'	Ullah et al. 2015
GAPDH	5'-TCCCTGAGCTGAACGGGAAG-3'	5'-GGAGGAGTGGGTGTCGCTGT-3'	Krock et al., 2017 & Cherif et al. 2019
GM-CSF	5'-TCTCAGAAATGTTTGACCTCCA-3'	5'-GCCCTTGAGCTTGGTGAG-3'	Ullah et al. 2015
GRO/ CXCL1	5'-TGAAGGCAGGGGAATGTATGTG-3'	5'-AGCCCCTTTGTTCTAAGCCA-3'	Bayo et al. 2017
IFN-γ	5'-AACTACTGATTTCAACTTCTC-3'	5'-ATTACTGGGATGCTCTT-3'	Huibers et al. 2011
IL-1β	5'-ACAGATGAAGTGCTCCTTCCA-3'	5'-GTCGGAGATTCGTAGCTGGAT-3'	Krock et al., 2017
IL-6	5'-TGAACCTTCCAAAGATGGCTG-3'	5'-CAAACTCCAAAAGACCAGTGATG-3'	Cherif et al., 2019
NGF	5'-AAGTGCCGGGACCCAAAT-3'	5'-TGAGTTCCAGTGCTTTGAGTCAA-3'	Krock et al., 2017
p16	5'-CTGCCCAACGCACCGAATA-3'	5'-GCTGCCCATCATCATGACCT-3'	Cherif et al. 2019
p21	5'-GAGACTCTCAGGGTCGAAAAC-3'	5'-GGCGTTTGGAGTGGTAGAAA-3'	Cherif et al. 2019
TGF-β	5'-TCCTGGCGATACCTCAGCAA-3'	5'-CTCAATTTCCCCTCCACGGC-3'	Aref-Eshghi et al. 2015
TLR-1	5'-CAGTGTCTGGTACACGCATGGT-3'	5'-TTTCAAAAACCGTGTCTGTTAAGAGA-3'	Krock et al., 2017

TLR-2	5’-GGCCAGCAAATTACCTGTGTG -3’	5’-AGGCGGACATCCTGAACCT-3’	Krock et al., 2017
TLR-4	5’-CAGAGTTTCCTGCAATGGATCA-3’	5’-GCTTATCTGAAGGTGTTGCACAT-3’	Krock et al., 2017
TLR-6	5’-GAAGAAGAACAACCCTTTAGGATAGC-3’	5’-AGGCAAACAAAATGGAAGCTT-3’	Krock et al., 2017
TNF-α	5’-ATGTTGTAGCAAACCCTCAAGC-3’	5’-TCTCTCAGCTCCACGCCATT-3’	Zhai et al. 2019
NF-L	5′-AGACATCAGCGCCATGCA-3′	5′-TTCGTGCTTCGCAGCTCAT-3′	Chung et al. 2010
VGF	5′-GCTCGAATGTCCGAAAACGT-3′	5′-ACACTCCTTCCCCGAACTGA-3′,	Chung et al. 2010
PVR	5′- ATGAGTGTCAGATTGCCACGTT-3′	5′- TCGGGCGAACACCTTCAG-3′	Chung et al. 2010
Plaur	5′- GGCTGGACCCAGGAACTTTT-3′	5′- CGCCTGTCCTCAAAGATGGA-3′	Chung et al. 2010
Plk2	5′- GCCCCACACCACCATCA-3′	5′-GGTCGACTATAATCCGCGAGAT-3′	Chung et al. 2010
