# Supplementary figures and images for: Senolytic Combination Treatment Is More Potent Than Single Drugs in Reducing Inflammatory and Senescence Burden in Cells from Painful Degenerating IVDs
Source: Biomolecules. 2023 Aug 16;13(8):1257. doi: 10.3390/biom13081257 (PMC10452201; doi:10.3390/biom13081257)

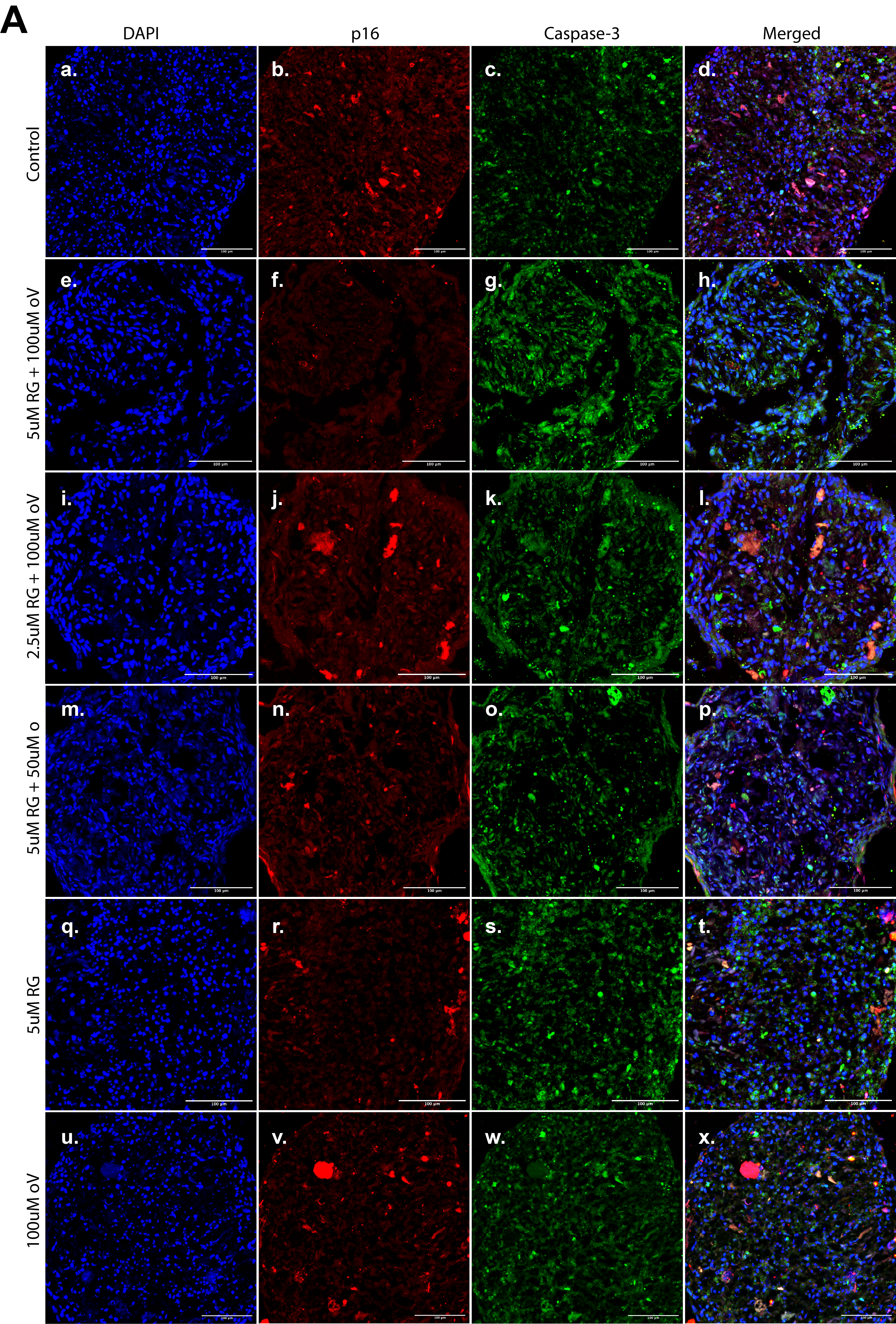

Supplement: Supplementary file 1 [file biomolecules-13-01257-s001.zip › Supplementary Figure S2.png]

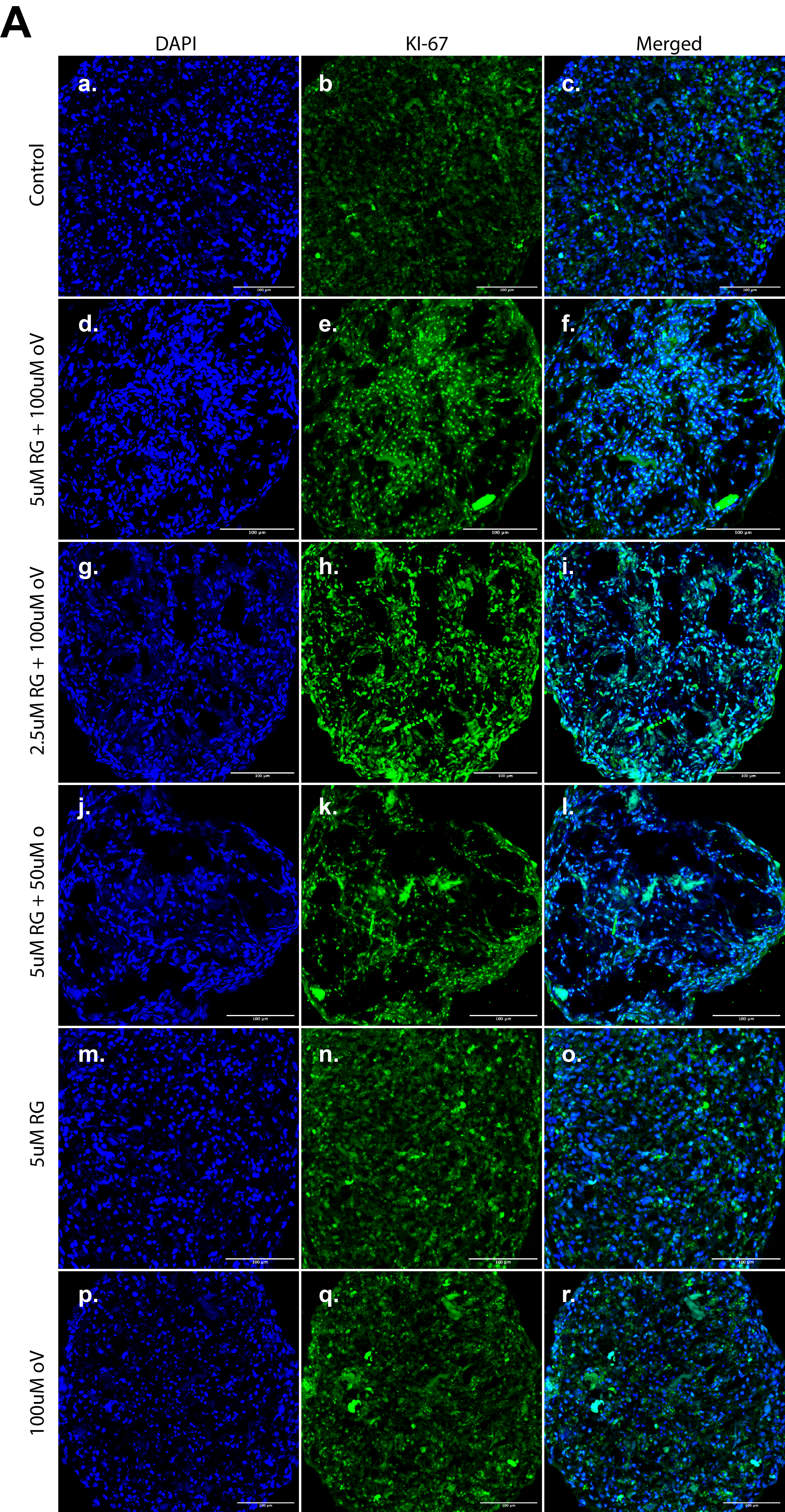

Supplement: Supplementary file 1 [file biomolecules-13-01257-s001.zip › Supplementary Figure S3.png]
